# Propyl Gallate Treatment Improves the Postharvest Quality of Winter Jujube (*Zizyphus jujuba* Mill. cv. Dongzao) by Regulating Antioxidant Metabolism and Maintaining the Structure of Peel

**DOI:** 10.3390/foods11020237

**Published:** 2022-01-17

**Authors:** Chao Wang, Cunkun Chen, Xiaoyang Zhao, Caie Wu, Xiaohong Kou, Zhaohui Xue

**Affiliations:** 1 School of Chemical Engineering and Technology, Tianjin University, Tianjin 300072, China; wangchao9582@163.com (C.W.); zxy467186123@163.com (X.Z.); zhhxue@tju.edu.cn (Z.X.); 2 National Engineering Technology Research Center for Preservation of Agricultural Products, Key Laboratory of Storage of Agricultural Products, Ministry of Agriculture and Rural Affairs, Tianjin Key Laboratory of Postharvest Physiology and Storage of Agricultural Products, Tianjin 300384, China; cck0318@126.com; 3 College of Light Industry and Food Engineering, Nanjing Forestry University, Nanjing 210037, China; wucaie@njfu.edu.cn

**Keywords:** winter jujube, propyl gallate, antioxidant enzyme activity, non-enzymatic antioxidant, peel microstructure

## Abstract

The quality and color of winter jujube fruits are easy to change after harvest. We studied the regulation mechanism of propyl gallate (PG) on post-harvest physiological quality of winter jujube, from the perspective of antioxidant metabolism and peel structure. In our research, winter jujube fruits were treated with 0.001 mol L^−1^ PG solution for 20 min. Our results showed that PG delayed the development of peel color, and improved the firmness, total soluble solids (TSS), and titratable acid (TA) of winter jujube. Meanwhile, the PG treatment had higher content of total phenols, total flavonoids, ascorbic acid (AsA), and reduced glutathione (GSH), and kept the enzyme activity including superoxide dismutase (SOD), catalase (CAT), ascorbate peroxidase (APX), and peroxidase (POD) at a higher level. PG treatment reduced membrane oxidative damage and maintained the integrity of pericarp structure by reducing electrolyte leakage (EL), lipoxygenase activity (LOX), hydrogen peroxide (H_2_O_2_), and malondialdehyde (MDA) content in the peel. Accordingly, PG improved the postharvest quality of jujube fruits by regulating antioxidant metabolism and maintaining the structure of peel. The appropriate concentration of PG has good application potential in the storage and preservation of fresh fruits such as winter jujube.

## 1. Introduction

Winter jujube (*Zizyphus jujuba* Mill. cv. Dongzao) has a long history of cultivation in China and is a famous fresh fruit. It is delicious and juicy, with thin and crisp peel, and rich in dietary fiber, amino acids, vitamin C, and minerals [1]. They are popular among consumers and have good market prospects. However, winter jujubes are prone to water loss, discoloration, softening, and loss of freshness during transportation, storage, and marketing, resulting in a rapid decrease in edible quality and nutritional value of the fruit [2,3]. Accordingly, how to keep the freshness of winter jujube fruits after harvest is an urgent problem to be solved during the industrialization of winter jujube.

The postharvest quality and consumer acceptability of fruits and vegetables are determined by their physicochemical properties and color. Physicochemical indicators of fruit are closely related to their storage quality, such as flavor, texture, and taste [4]. Color is the main criterion for calculating the maturity and freshness of fruits and vegetables. Our previous research indicated that the reddening of winter jujube peel is related to the metabolism of anthocyanins and flavonoids, among which anthocyanins play a major role [5]. One of the causes of pericarp browning is enzymatic browning, where phenolic substrates are oxidized to colored quinones catalyzed by oxidase [6]. The authors of [7] pointed out that the compartmentation of plant cells was damaged by membrane lipid peroxidation metabolism, providing conditions for phenolic substrate to contact with oxidative enzymes and undergo enzymatic browning. Reactive oxygen species (ROS) accumulation can result in oxidative damage to the membrane structure of cells, indirectly promoting membrane lipid peroxidation and disrupting the fluidity and functionality of the cell membrane [8]. Our previous research found that the microstructure changes on the winter jujube peel during cold storage may promote the accumulation of quinones in pericarp [9]. Based on the above research, the ROS metabolism, membrane lipid peroxidation metabolism, and peel structure may serve as a common regulatory mechanism for fruit to maintain postharvest quality.

Propyl gallate (PG) is increasingly used for the preservation of several fruits and vegetables due to its antioxidant properties and economy. PG belongs to the family of phenolic compounds with free radical scavenging capacity [10]. Recently, the research reported that PG reduces longan (*Dimocarpus* longan Lour.) ROS accumulation, inhibits the degree of membrane lipid peroxidation, thereby improving its postharvest quality [11,12]. This result indicates that PG has the potential to regulate postharvest physicochemical properties of fresh fruits. Nevertheless, the potential of PG in controlling postharvest quality of winter jujube fruits and its regulatory mechanisms remain unclear.

In light of the above considerations, the changes in the winter jujube fruits treated PG, including physiochemical indicators, peel structure, ROS production, malondialdehyde (MDA) content, cell membrane permeability, non-enzymatic antioxidants content, and activities of antioxidant enzymes were investigated. This work aimed at assessing the regulation mechanism of PG on the post-harvest physiological quality of winter jujube via the analysis of antioxidant metabolism and peel structure. Meanwhile, we sought to validate the effectiveness of PG treatment as an economical and safe method on the quality maintenance of winter jujube after harvest.

## 2. Materials and Methods

### 2.1. Materials

The winter jujube fruits (soluble solids content: 7–8%; firmness: 15–17 N) were selected in the local orchard in Huanghua, Hebei Province, China (117°32′ E, 38°46′ N), and transported to the laboratory immediately. The fruits were selected on the basis of uniform color and size, and any blemished or diseased fruits were excluded. PG was purchased from HEOWNS Technology Co., Ltd. (Tianjin, China).

### 2.2. Postharvest Treatments

According to the previous study [11] and our preliminary experiments, the results showed that the post-harvest quality and freshness of winter jujube fruits were significantly improved by 0.001 mol L^−1^ PG aqueous solution. Accordingly, 0.001 mol L^−1^ was chosen as the final treatment concentration.

All fruits were divided into two groups, randomly, (three replicates per group, 350 fruits per replicate) for control and PG treatment. The groups were as follows: (1) control (deionized water) and (2) PG treatment (0.001 mol L^−1^ PG aqueous solution). All fruits were immersed for 20 min and then air-dried for 2 h, the two groups were put in polyethylene (0.35 m × 0.25 m, 0.007 mm) bags (50 fruits per bag), respectively. Then, they were stored at 80–85% humidity and 0 °C for 90 d. The 150 fruits (three bags) were randomly selected from the control and PG treatment for the evaluation on the peel structure and the analysis of physiochemical quality attributes. The Liquid nitrogen was used to treat peels (1–2 mm thick), and those were placed at −80 °C for subsequent indicators determination.

### 2.3. Reddening Index, Reddish Peel Area, Chromatic Value, Firmness, Total Soluble Solids (TSS), and Titratable Acid (TA)

Reddening index was evaluated according to the reported method [9], with some modifications. Fifty fruits were evaluated based on reddish peel area by the following visual appearance scale: 0 = fruit has no reddish peel, 1 = 0–25%, 2 = 25–50%, 3 = 50–75%, 4 = 75–100%, 5 = 100%. The equation of the reddening index was as follows:

(1) $$\mathrm{Reddening}\;\mathrm{index}\;\left(\%\right)=\frac{\sum^{\text{​}}\left(\mathrm{reddening}\;\mathrm{scale}\times \mathrm{number}\;\mathrm{of}\;\mathrm{fruit}\;\mathrm{in}\;\mathrm{each}\;\mathrm{scale}\right)}{\left(5\times 50\right)}\;$$

Reddish peel area of peel was measured using ImageJ software (National Institutes of health, Bethesda, MD, USA). Chromatic values were determined with a Chroma Meter (Osaka, Japan) at the equator of the fruits and it was based on the average of three measurements.

Firmness was determined with a GY-1 fruits firmness meter (Mudanjiang Machinery Research Institute, Heilongjiang, China) at the equator of the fruits. Firmness is based on the average of three measurements, reported as newtons (N).

TSS content was determined in the light of [5], with slight modifications. Pulp (2 g) was well mixed with 2 mL deionized water and then filtered. The filtrate was determined with a PAL-1 handheld refractometer (ATAGO, Japan). The TSS content was reported as %.

TA content was measured as reported by Sogvar et al. [13], with slight modifications. The aliquots (20 mL) were titrated to pH 8.1 with 0.01 mol L^−1^ NaOH and reported as %.

### 2.4. The Microstructure and Ultrastructure of Winter Jujube Peel

The pericarp microstructure was observed following the method of [14], with some modifications. Peels (0.5–1.0 cm^2^) were fixed in 2.5% pentanediol for 2 h (25 °C), and overnight at 4 °C and then fixed in 1% osmic acid for 2 h. Each sample was dehydrated with 30, 50, 70, 80, 90, 95, and 100% ethanol for 15 min, then were placed in a vacuum freeze-dryer for 24 h. The samples were sprayed with gold using a Cressington 108Auto ion sputterer, and then observed under Regulus 8100 SEM (Hitachi, Japan).

The pericarp ultrastructure was observed following the method of Zhao et al. [15], with some modifications. Peels (1 cm^3^) were fixed with 2.5% pentanediol over night at 4 °C, then were fixed in 1% osmium acid (prepared with 0.1 mol L^−1^, pH 7.5 phosphate buffer) for 7 h. Each sample was dehydrated with the same conditions as above. Different embedding agents were used to infiltrate the sample, including acetone: embedding medium 812 (*v*/*v* = 1:1, 24 h), acetone: embedding medium 812 (*v*/*v* = 1:3, 4 h) and pure embedding medium 812 (24 h). Afterwards, the samples were heated (37 °C) for 12 h, and then heating (60 °C) for 48 h. The ultrathin (70 nm thick) of the resin blocks were cut with a Leica UC7 ultra-thin microtome, and then dyed (2.6% lead citrate, 2% uranyl acetate) for 8 min, respectively. Observation was performed with JEM-1400 Flash TEM (JEOL, Tokyo, Japan).

### 2.5. Electrolyte Leakage (EL), MDA, Lipoxygenase (LOX) and H_2_O_2_

EL was measured following the method of [7], with some modifications. Twenty peel discs (diameter: 8 mm, thickness: 1 mm) from 10 fruits were placed in 40 mL deionized water. The determination of EL by a DDS-11A conductivity meter (Yidian Scientific Instruments Co., Ltd., Shanghai, China). Record the following measured values: room temperature (P_0_), stand for 10 min (P_1_), boil for 15 min and then cool to room temperature (P_2_). The equation of EL was as follows:

(2) $$\mathrm{EL}\;\left(\%\right)=\frac{\left(P_{1}-P_{0}\right)}{\left(P_{2}-P_{0}\right)}\times 100\%\;$$

MDA content was determined following the method of Kou et al. [5], with some modifications. Peel (1 g) was mixed with 10 mL of 10% trichloroacetic acid (TCA), centrifuged (4 °C, 12,000× *g*) for 10 min. 2 mL supernatant was mixed with 2 mL of 6.7 g L^−1^ thiobarbituric acid (TBA) and heated at 100 °C for 15 min. Absorbance was read at 532, 600, and 450 nm against a blank. The MDA content was reported as μmol g^−1^ fresh weight (FW).

LOX activity was assayed by using the methods reported [16,17], with some modifications. Peel (1 g) was mixed with 10 mL of 0.05 mol L^−1^, pH 7.0, phosphate buffer solution (PBS), containing 1% Triton X-100 and 4% polyvinyl pyrrolidone (PVP), centrifuged (4 °C, 12,000× *g*) for 15 min. The reaction mixture included sample enzyme extract (0.2 mL), 0.1 mol L^−1^ acetic acid–sodium acetate solution (2.75 mL), 0.1 mol L^−1^ sodium linoleate solution (0.05 mL) and then incubated for 10 min at 30 °C. A change of 0.001 at absorbance per min at 234 nm was defined as one unit of LOX activity (U). Protein content was measured following the method of Bradford [18]. All enzyme activity units were reported as U mg^−1^ protein.

H_2_O_2_ content was assayed by the method reported previously [19], with some modifications. Peel (1 g) was mixed in cold acetone (10 mL), centrifuged (4 °C, 12,000× *g*) for 20 min. The supernatant (1 mL) was mixed with reaction solution (0.1 mL of 20% TiCl_4_, 0.2 mL of NH_3_·H_2_O), and then re-centrifuged. The cold acetone was used to wash the precipitate repeatedly and dissolved in 3 mL of H_2_SO_4_ (2 mol L^−1^) and then re-centrifuged. The absorbance at 415 nm was reported. The H_2_O_2_ content was reported as mmol g^−1^ FW.

### 2.6. Total Phenols, Total Flavonoids, Ascorbic Acid (AsA), and Reduced Glutathione (GSH)

The extraction mixture that included peel (2 g), 80% cold methanol (20 mL) were ultrasonic for 10 min, centrifuged (4 °C, 12,000× *g*) for 10 min. All supernatant was collected by repeating the above steps and then was concentrated by vacuum at 45 °C. The concentrated solution was collected and standardized to 10 mL with methanol.

Total phenol content was measured following the previous methods [20,21], with some modifications. The reaction system consists of 0.125 mL of extracting solution and 0.125 mL of Folin–Ciocalteu reagent. After 6 min, 1.25 mL of 7% Na_2_CO_3_ and 8.5 mL of distilled water was added to the mixture. Then, the mixture was stationary for 90 min. The absorbance at 760 nm was reported. The total phenol content was reported as mg g^−1^ FW.

Total flavonoids content was measured following the method [22], with some modifications. The 0.75 mL of 5% NaNO_2_ was mixed with extracting solution (1 mL), and then incubated for 5 min. The 10% Al (NO_3_)_3_ (0.5 mL) and 5% NaOH (4 mL) was added to the mixture. The volume of mixture was adjusted to 10 mL by distilled water. The absorbance at 510 nm was recorded. The total flavonoids content was calculated using an equation from the standard curve, and the content is reported in mg g^−1^ FW.

AsA and GSH content was measured following the method of Ge et al. [23], with some modifications. Peel (1 g) was mixed with 5% cold TCA (10 mL) and centrifuged (4 °C, 12,000× *g*) for 20 min. The supernatant was collected. For AsA, the reaction mixture contained of supernatant (1 mL), 5% TCA (1 mL), anhydrous ethanol (1 mL), 4% phosphoric acid (0.5 mL), 5% red phenanthroline (1 mL), 0.3% ferric chloride (0.5 mL). The absorbance at 534 nm was measured. The AsA content is represented in mg g^−1^ FW. For GSH, the mixture, 1 mL of supernatant, 1 mL of 0.1 mol L^−1^ PBS (pH 8.0), and 0.5 mL of 0.004 mol L^−1^ o-nitrobenzoic acid, was incubated at 25 °C for 10 min. The absorbance at 412 nm was recorded. GSH content is reported in mmol g^−1^ FW.

### 2.7. Superoxide Dismutase (SOD), Catalase (CAT), Ascorbate Peroxidase (APX), and Peroxidase (POD) Activity

Activity of SOD, CAT, APX, and POD was determined according to the previous methods [11,24], with some modifications. Peel (1 g) was mixed with 10 mL of 50 mmol L^−1^, pH 7.0, PBS (containing 50 g L^−1^ PVP, 5 mmol L^−1^ dithiothreitol, 1 mmol L^−1^ polyethylene glycol), centrifuged (4 °C, 12,000× *g*) for 20 min. The supernatant was pooled and placed at 4 °C.

For SOD, 0.1 mL of extracting solution and 2.9 mL of reaction solution: 0.013 mol L^−1^ methionine, 0.075 mol L^−1^ nitroblue tetrazolium (NBT), 0.01 mmol L^−1^ EDTA-Na_2_, 0.002 mmol L^−1^ riboflavin, were mixed and then irradiated at 4000 Lx for 15 min. The unirradiated solution as control and the absorbance at 560 nm was read. The amount of enzyme that would inhibit 50% photoreduction of NBT was defined as one unit of enzyme activity (U). 

For CAT, the reaction system included extracting solution (0.1 mL) and 0.02 mol L^−1^ H_2_O_2_ (2.9 mL) prepared with 50 mmol L^−1^ PBS. A change of 0.01 at absorbance per min at 240 nm was defined as one unit of CAT activity (U).

For APX, the extracting solution (0.1 mL) was mixed with reaction mixture (2.9 mL), and the reaction mixture contained 0.5 mmol L^−1^ ascorbic acid, 0.1 mmol L^−1^ EDTA-Na_2_ and 0.1 mmol L^−1^ H_2_O_2_. The amount of enzyme that oxidized 0.001 mol L^−1^ ascorbate per min at 290 nm was defined as one unit of APX activity (U).

For POD, the reaction mixture consisted of extracting solution (0.8 mL), 0.025 mol L^−1^ guaiacol solution (1.1 mL) and 0.5 mol L^−1^ H_2_O_2_ (0.1 mL). The absorbance at 470 nm was recorded. A change of 0.1 at absorbance per min at 470 nm was defined as one unit of POD activity (U).

### 2.8. Statistical Analysis

The experimental results are expressed as mean ± standard error (SE), based on three replicates, and the analysis of all results were executed using the IBM SPSS Statistics 26.0 (SPSS Inc., Chicago, IL, USA) software. The two-way analysis of variance (ANOVA) was used to assess the effect of treatments (factor A) and storage times (factor B) and their interaction on each response variable, in which factor A had two levels (CK, PG treatment) and factor B had seven levels (Day 0, Day 15, Day 30, Day 45, Day 60, Day 75, Day 90). Duncan’s test was used to compare the means between CK and PG treatment at the same storage time, at the significance level (*p* < 0.05 or *p* < 0.01).

## 3. Results

### 3.1. Changes of Visual Appearance, Reddish Peel Area, and Chromatic Value in the Winter Jujube Fruit after PG Treatment

**Figure 1:**
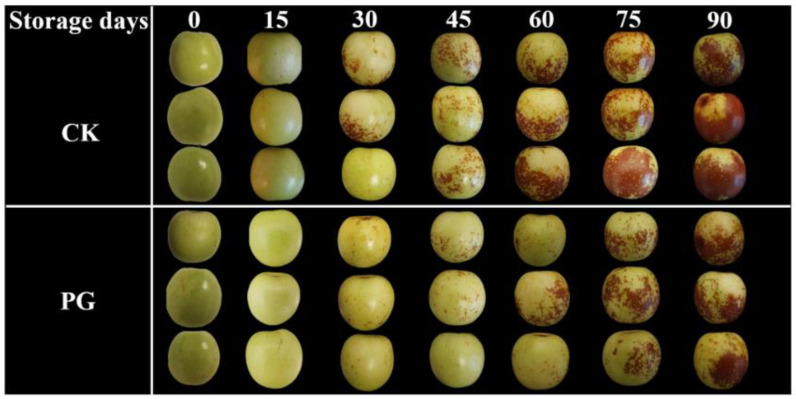
Effects of PG treatment on the visual appearance of winter jujube fruits.

The visual appearance of winter jujube fruits in both groups almost remained unchanged in the initial fifteen days of storage (Figure 1), but the peel of the control turned red from Day 30, 15 d earlier than that of PG treatment. The determination results of reddish peel area of Figure 1 using ImageJ software and chromatic value of peel were provided in (supplementary Figures S1 and S2). PG treatment significantly delayed the development of pericarp color of winter jujube during the storage period, compared with the control.

### 3.2. Results of Two-Way Analysis of ANOVA

The results of the effect of treatments and storage times and their interaction on each response variable via two-way analysis of ANOVA are shown in Table 1. Except for TSS, the extremely significant differences on treatments were observed for all variables. Meanwhile, there were extremely significant differences for all variables in storage time and the interaction between treatments and storage time. In addition, the F values of treatments for all variables were higher than the F values of storage time, except the reddening index, firmness, TSS, EL, and total phenol, which indicated that the effect of treatments on postharvest quality of winter jujube was more significant than the storage times.

### 3.3. Changes of Reddening Index, Firmness, TSS, and TA in the Winter Jujube Fruit after PG Treatment

**Figure 2:**
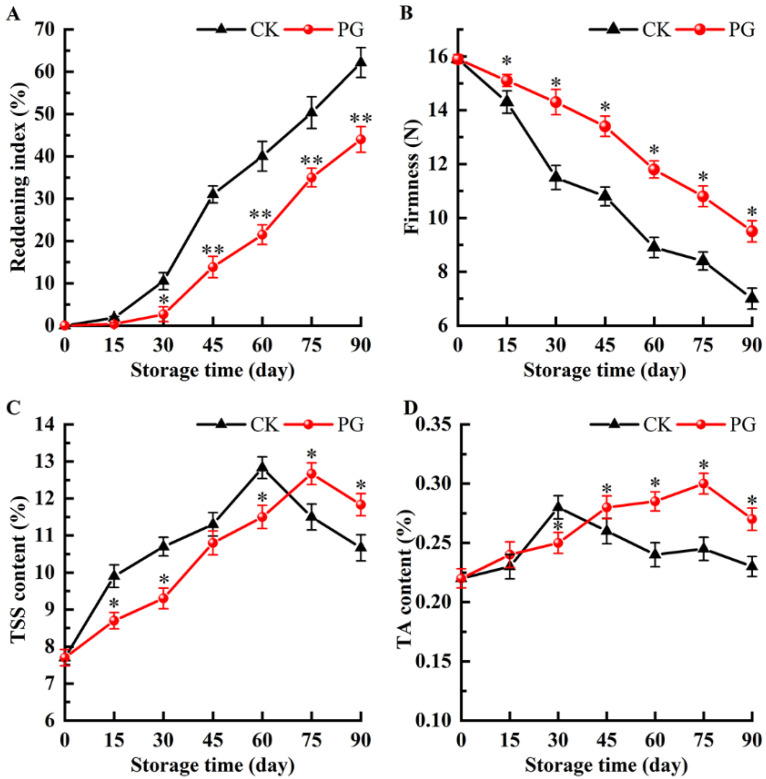
Effects of PG treatment on reddening index (**A**), firmness (**B**), total soluble solids (TSS) (**C**), and titratable acid (TA) (**D**) of winter jujube fruits. Vertical bars represent the SE of the mean. The asterisks indicate significant difference between two treatments during the same storage period (* *p* < 0.05, ** *p* < 0.01).

Figure 2A shows that the reddening index gradually increased during storage, which was consistent with Figure 1. PG treatment maintained higher firmness during storage compared with control (Figure 2B). The content of TSS and TA in peel of control reached the peak value on day 60 and 30, respectively, while PG treatment delayed the time to reach the peak value for TSS and TA. (Figure 2C,D).

### 3.4. Changes of Microstructure and Ultrastructure in the Winter Jujube Peel after PG Treatment

**Figure 3:**
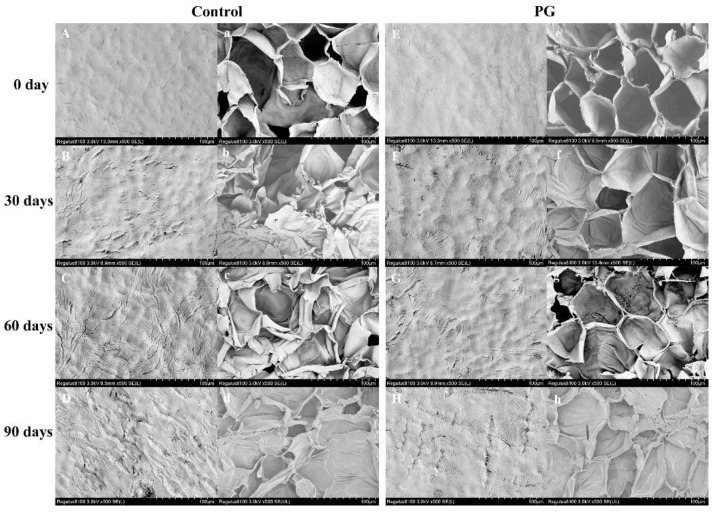
Effects of PG treatment on peel microstructure of winter jujube. Control (**A–D**,**a–d**), PG treatment (**E–H**,**e–h**). Storage for 0 d (**A**,**a**,**E**,**e**), storage for 30 d (**B**,**b**,**F**,**f**), storage for 60 d (**C**,**c**,**G**,**g**), storage for 90 d (**D**,**d**,**H**,**h**). Magnification: 500, bar = 100 μm.

Figure 3A,E shows that the peel surface on Day 0 with a relatively rough with a few micro-cracks, and the endocarp cells are irregularly shaped but closely packed (Figure 3A,E). The number and gap of micro-cracks were gradually increased on the control exocarp, and its inner peel also collapsed during storage (Figure 3B–D,b–d), whereas the PG treatment had a more complete structure of exocarp and endocarp during the same period (Figure 3F–H,f–h).

**Figure 4:**
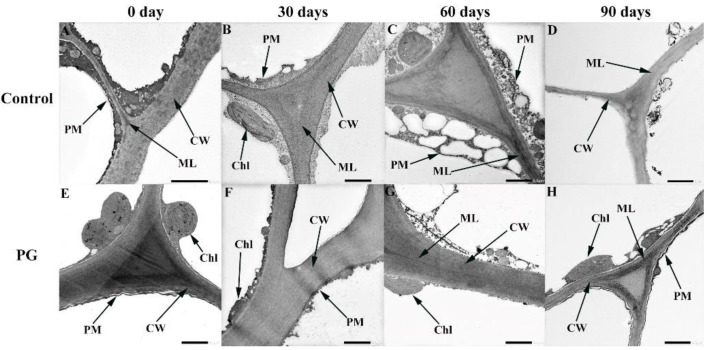
Effects of PG treatment on peel ultrastructure of winter jujube. Control (**A**–**D**), PG treatment (**E**–**H**). Stored for 0 d (**A**,**E**), stored for 30 d (**B**,**F**), stored for 60 d (**C**,**G**), stored for 90 d (**D**,**H**). Magnification: 3000, bar = 2.0 μm. Chloroplast (Chl), middle layer (ML), plasma membrane (PM), cell wall (CW).

As shown in Figure 4, on Day 0, the pericarp plasma membrane was tightly connected with the cells (Figure 4A,E). The cell wall structure of control worsened firstly and the degree of degradation increased gradually (Figure 4B–D). Nevertheless, the cell wall structure in the fruits treated with PG solution was comparatively complete and preserved at the same storage period. However, the cell wall structure was relatively complete and preserved in the fruits treated with PG solution (Figure 4F–H).

### 3.5. Changes of EL, H_2_O_2_, LOX, and MDA in the Winter Jujube Peel after PG Treatment

**Figure 5:**
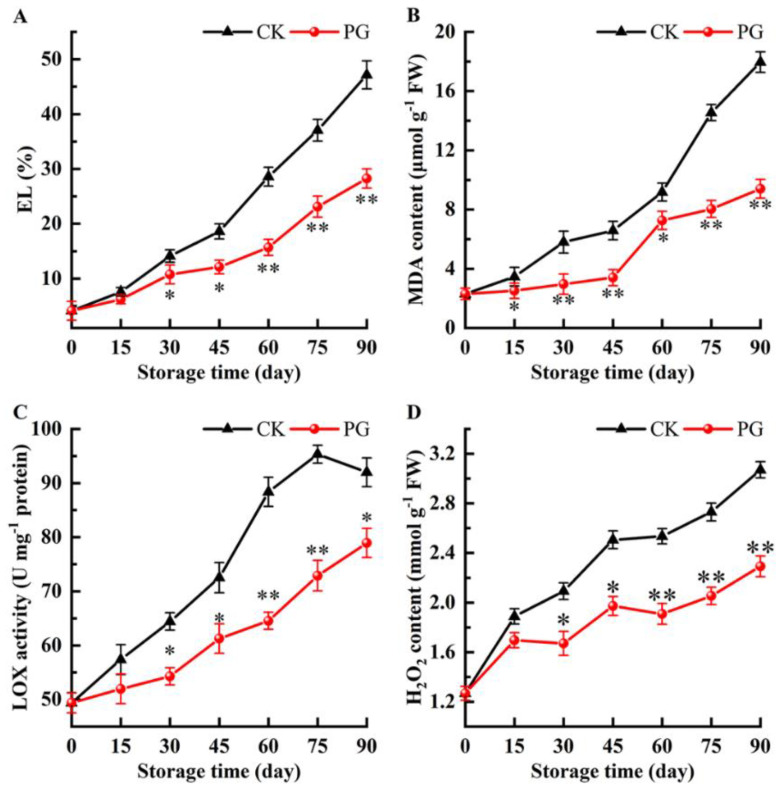
Effects of PG treatment on electrolyte leakage (EL) (**A**), malondialdehyde (MDA) content (**B**), lipoxygenase (LOX) activity (**C**), and hydrogen peroxide (H_2_O_2_) content (**D**) in the peel. Vertical bars represent the SE of the mean. The asterisks indicated significant difference between two treatments during the same storage period (* *p* < 0.05, ** *p* < 0.01).

The EL and MDA of winter jujube fruits had a similar pattern and gradually increased during cold storage (Figure 5A,B). Both parameters were significantly decreased by the PG treatment throughout the storage period. On Day 90, EI and MDA in the fruits treated with PG were 40.11% and 47.63%, respectively, lower than that in the control fruits. 

As shown in Figure 5C, LOX activity in the peel of control fruits increased gradually and decreased after reaching the peak value (Day 75). Although the LOX activity in peel of PG-treated fruits showed the same change trend, its value was significantly lower than that of the control.

The H_2_O_2_ accumulation in winter jujube fruits increased gradually, but the H_2_O_2_ content of PG treatment was significantly lower than that of control except for Day 15. For instance, the H_2_O_2_ content of peel was 25.41% lower than in control fruits 90 d after PG treatment (Figure 5D).

### 3.6. Changes of Total Phenols, Total Flavonoids, AsA, and GSH in the Winter Jujube Peel after PG Treatment

**Figure 6:**
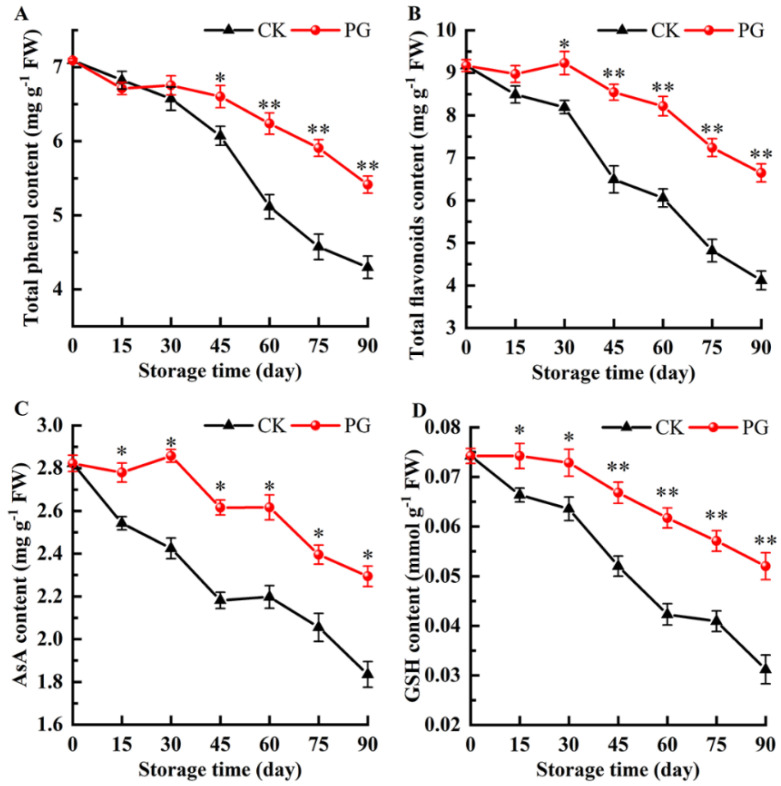
Effects of PG treatment on total phenols (**A**), total flavonoids (**B**), ascorbic acid (AsA) (**C**), and reduced glutathione (GSH) (**D**) in the peel. Vertical bars represent the SE of the mean. The asterisks indicate significant difference between two treatments during the same storage period (* *p* < 0.05, ** *p* < 0.01).

There was a downtrend of total phenols content (Figure 6A). Total phenols content in the control fruits was lower compared with that of the PG-treated fruits from storage Day 45 to Day 90. Similarly, the total flavonoids content in the control fruits kept decreasing constantly during storage. The remarkable higher total flavonoids content was found in the treatment fruits from Day 30 to Day 90 (Figure 6B).

The AsA content in the pericarp of control jujube fruits declined gradually, whereas the PG-treated fruits exhibited a significant higher content of the ASA (Day 90), which was 19.65% higher than the control (Figure 6C). The GSH content in the jujube fruits had a similar pattern during cold storage. Likewise, the GSH content in the PG-treated fruits was 40.38% higher than the control after Day 90 storage (Figure 6D).

### 3.7. Changes of the Activity of SOD, CAT, APX, and POD in the Winter Jujube Peel after PG Treatment

**Figure 7:**
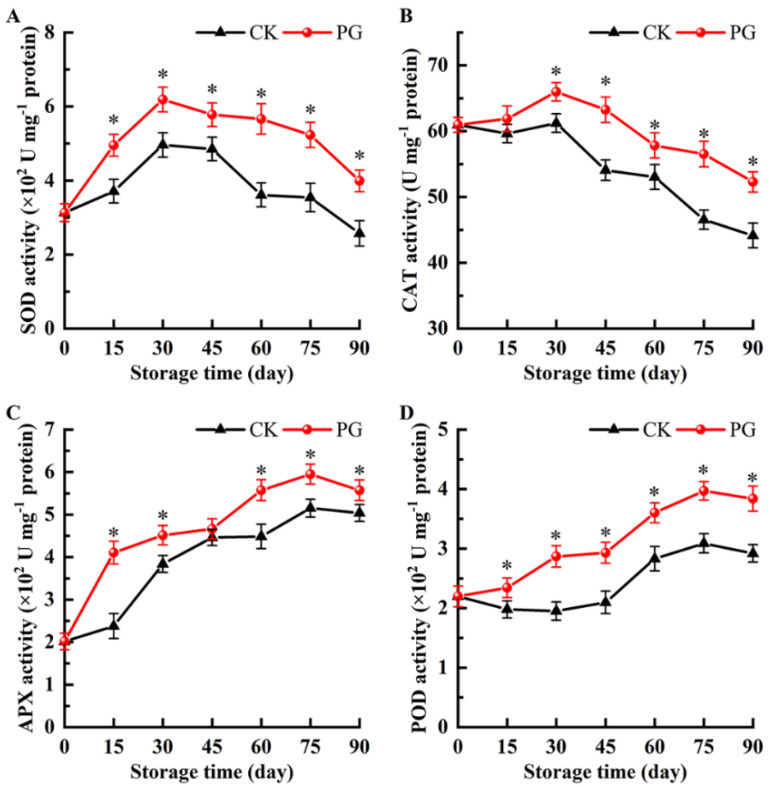
Effects of PG treatment on the activity of superoxide dismutase (SOD) (**A**), catalase (CAT) (**B**), ascorbate peroxidase (APX) (**C**), and peroxidase (POD) (**D**) in the peel. Vertical bars represent the SE of the mean. The asterisks indicate significant difference between two treatments during the same storage period (* *p* < 0.05).

The SOD activity of the two treatments increased first and then decreased, and reached the peak on Day 30, while the SOD activity in the peel of PG-treated fruits was 24.8% higher than the control (Figure 7A). In addition, SOD activity of PG treatment was significantly higher than the control at other monitoring time point.

The variation trend of CAT activity was consistent with SOD activity (Figure 7B). Meanwhile, the CAT activity in the peel of PG-treated fruits reached 65.98 U mg^−1^ protein (Day 30), which was 7.21% higher than the control fruits. In addition, except Day 15, the CAT activity of PG treatment was higher than the control during storage.

During storage, the activity of APX in the peel of PG-treated fruits was significantly higher than the control except Day 45, which were 41.95%, 15.04%, 19.39%, 13.45%, and 9.52% higher than the control, respectively (Figure 7C).

As demonstrated in Figure 7D, the POD activity maintained at a higher level in the peel of PG-treated fruits, but decreased slightly from Day 75. POD activity in the control fruits was 1.18-, 1.47-, 1.40-, 1.27-, 1.28-, and 1.32-times lower than that of the fruits with PG treatment during storage.

## 4. Discussion

Reddening index, firmness, TSS, and TA are important physicochemical properties for assessing postharvest quality of fruits. Reddening index and firmness are the essential attributes in determining consumer preference and storability of winter jujube [9,25]. TSS and TA are considered to be the main indicators to evaluate the taste, flavor, and quality of postharvest jujube fruits [26]. In this study, the firmness of the postharvest winter jujube decreased progressively, while the reddening index increased. At the same time, the content of TSS and TA had a similar trend of change in the winter jujube, i.e., increase first and then decrease (Figure 2). This result was consistent with the report that the postharvest treatments by nitric oxide or composite film to prolong the storage of winter jujube fruits [3,9]. However, the increase in reddening index and TSS were delayed by PG treatment, and maintained the higher firmness and TA, which indicated that the capacity of PG to improve the quality of postharvest winter jujube can be connected with maintaining of physicochemical properties. The results were consistent with previous studies on longan [12], korla fragrant pear [27], and melon [28].

The microstructure of the surface of fruits and vegetables has an important influence on their firmness, moisture, and strength of respiration metabolism [29,30]. Previous research proved that the structure of the peel is not only a parasitic site for pathogenic microorganisms, but also a natural barrier against abiotic stress and insect damage [31,32]. The above result indicates that the peel structure is crucial to the quality of fruits after harvest. The authors of [33] reported that the peel structure of postharvest mango (*Mangifera indica* L.) can reflect the integrity of the cell membrane, and pointed out that this may be related to lipid peroxidation metabolism and the accumulation of ROS. In addition, the further study showed that the increase of LOX activity, ROS and MDA content disrupted the dynamic balance of membrane permeability in pericarp, aggravating membrane lipid peroxidation, and then accelerated the destruction of structure on the pericarp cell wall [34]. In our study, the microstructure of jujube peel deteriorated during storage; however, the results showed that the damage degree in the control fruits was higher than the PG treatment. This indicates that PG treatment may maintain the integrity of the peel structure by regulating the accumulation of ROS and the degree of membrane lipid peroxidation in the jujube peel [11,12]. The winter jujube cannot rely on its own physiological metabolic activities to delay aging, softening, and the rate of nutrient loss at harvest. Therefore, peel is a natural barrier for jujube fruits to contact the external environment, it is of great significance to maintain the postharvest quality and nutrition. The above findings showed that the winter jujube treated with PG had more complete pericarp structure, which played a positive role in maintaining the quality and nutritional composition of winter jujube. The authors of [34,35] reported that are consistent with our research results.

Low temperature stress causes changes in cell membrane fluidity and integrity, and EL is usually used as an indicator of cell membrane integrity [36]. MDA is often used to reflect the degree of lipid peroxidation in cell membranes [37]. It is well known that LOX is an important enzyme in the process of membrane lipid peroxidation, which disrupts the structure of phospholipid bilayer by catalyzing polyunsaturated fatty acids (linoleic acid, linolenic acid), increases the permeability of cell membrane and decreases the fluidity of cell membrane [17,38]. The content of ROS in fruits and vegetable tissues changes constantly during postharvest storage, which to cope with the impact of environmental stress on their metabolic activities. For instance, the low concentrations of ROS can act as signaling molecules to trigger defense mechanisms in fruits and vegetables; however, excessive ROS accumulation will cause oxidative damage to cell membrane, and then destroy the compartmentalization structure of plant cells [39,40]. H_2_O_2_ is an important ROS in higher plants with unique effects on effecting storage quality of fruits and vegetables [41]. In this study, the PG-treated fruits had lower content of MDA and H_2_O_2_ (Figure 5B,D), which can be related to the higher content of LOX, AsA, and GSH in the PG treatment (Figure 5C and Figure 6C,D). We speculated that PG may reduce the damage of membrane lipid peroxidation, slow down membrane lipid damage by maintaining high non-enzymatic antioxidant content (AsA, GSH) and LOX activity in pericarp (Figure 5D), thus protecting the structure and function of pericarp membrane (Figure 5A).

Polyphenols and flavonoids are both phenolic compounds. They can protect cells from oxidative damage caused by free radicals and prevent the initiation and reaction of oxidative chains to help membrane lipids resist peroxidation [21,42]. The AsA–GSH cycle runs in the chloroplast, mitochondria, and cytoplasm of plant cells, and it is the main H_2_O_2_ scavenging system [43]. AsA is not only a nutrient in fruits and vegetables, but also an endogenous antioxidant. AsA can decompose H_2_O_2_ into H_2_O and O_2_ under the catalysis of APX, and then under the action of GSH, the generated dehydroascorbic acid will be reduced to AsA [44]. ROS scavenging activity is enhanced in plants by maintaining higher content of non-enzymatic antioxidants [45]. In this study, PG treatment could slow down the decline of non-enzymatic antioxidant (Figure 6), which caused the lower content of MDA and H_2_O_2_ in the PG-treated fruits (Figure 5). The authors of [46] investigated the regulatory mechanism of PG treatment on post-harvest longan fruits quality and pulp degradation, and the results showed that PG treatment had the ability to remove ROS free radicals and preserve membrane structural integrity, which provided theoretical basis for our results. Our study provided evidence for a potential mechanism by which postharvest PG treatment activates the ASA–GSH cycle and non-enzymatic antioxidant system activity in the peel of Winter jujube. Therefore, this finding further validated our hypothesis about the effecting mechanism of postharvest PG treatment on the structural integrity of winter jujube pericarp.

Antioxidant enzymes are the main components of the cellular defense system to resist oxidative stress, and have the effect of helping plants resist peroxidation [38]. Among various antioxidant enzymes, SOD can eliminate superoxide anion radicals, CAT and POD can catalyze the decomposition of H_2_O_2_ into H_2_O and O_2_, and APX also has the function of decomposing H_2_O_2_, but unlike CAT, APX may be responsible for the fine regulation of ROS signal [47]. In our research results, compared with the control, the treatment with PG enabled winter jujube to have higher antioxidant enzyme activity during the entire storage period (Figure 7). We speculate that this may be related to the molecular structure of PG. As a polyphenolic antioxidant, the number and arrangement of phenolic hydroxyl groups on the benzene ring of PG represent its antioxidant and free radical scavenging ability [48]. In the pericarp cells of jujube, the phenolic hydroxyl group of PG released a large number of hydrogen atoms, and the positively charged hydrogen ions neutralized the negatively charged ROS free radicals in the pericarp cells, which weakened the diffusion degree of free radicals in the membrane lipid oxidation process, thereby improving the antioxidant of winter jujube.

## 5. Conclusions

It is clear that postharvest application of PG delayed senescence, and effectively maintained the winter jujube quality. The increased quality of jujube fruits after harvest by PG treatment might be associated with the integrity of peel structure and an increase in antioxidant capacity. Our results suggest that this was attained through an enhanced level of non-enzymatic antioxidants (total phenols, total flavonoids, AsA and GSH), and the decreased content of MDA, H_2_O_2_ as well as LOX activity, and the maintained activity of SOD, CAT, APX, POD, and the preservation of peel microstructure. This research may help further elucidate the mechanism underpinning PG-mediated preservation of postharvest winter jujube fruits quality. Overall, this research provided a theoretical basis for elucidating the potential mechanism underpinning the PG-mediated preservation of postharvest winter jujube quality.

## Figures and Tables

**Table 1:** Two-way ANOVA of treatments and storage times for winter jujube after harvest.

Variable	Treatments (A)	Storage Times (B)	Interaction (A × B)
	F	F	F
Reddening index	303.061 **	603.204 **	22.112 **
Firmness	148.836 **	163.285 **	6.755 **
TSS	4.605 *	81.618 **	9.595 **
TA	93.143 **	48.421 **	26.053 **
EL	262.602 **	341.015 **	29.288 **
MDA	438.375 **	404.521 **	50.161 **
LOX	86.234 **	66.957 **	5.974 **
H_2_O_2_	137.947 **	79.388 **	7.208 **
Total phenol	111.385 **	127.260 **	15.868 **
Total flavonoids	183.257 **	94.247 **	11.512 **
ASA	621.014 **	226.845 **	21.975 **
GSH	264.355 **	141.382 **	12.812 **
SOD	165.602 **	54.523 **	6.664 **
CAT	152.018 **	86.204 **	9.528 **
APX	96.392 **	169.214 **	8.785 **
POD	684.640 **	297.156 **	27.418 **

Total soluble solids (TSS), titratable acid (TA), electrolyte leakage (EL), malondialdehyde (MDA), lipoxygenase (LOX), ascorbic acid (AsA), reduced glutathione (GSH), superoxide dismutase (SOD), catalase (CAT), ascorbate peroxidase (APX), peroxidase (POD), * *p* < 0.05, ** *p* < 0.01.

## Data Availability

Not applicable.

## Supplementary Materials

The following are available online at https://www.mdpi.com/article/10.3390/foods11020237/s1, Figure S1: Effects of PG treatment on the reddish peel area of the peel. Vertical bars represent the SE of triplicate assays. The asterisks indicated significant difference between the control and PG-treated fruits during the same storage period (* *p* < 0.05, ** *p* < 0.01), Figure S2: Effects of PG treatment on the L*(A), a* (B) and b* (C) of winter jujube fruits. Vertical bars represent the SE of the mean. The asterisks indicated significant difference between two treatments during the same storage period (* *p* < 0.05, ** *p* < 0.01).
