# Supplementary material for: Propyl Gallate Treatment Improves the Postharvest Quality of Winter Jujube (Zizyphus jujuba Mill. cv. Dongzao) by Regulating Antioxidant Metabolism and Maintaining the Structure of Peel
Source: Foods. 2022 Jan 17;11(2):237. doi: 10.3390/foods11020237 (PMC8775024; doi:10.3390/foods11020237)
Supplement: Supplementary file 1 [file foods-11-00237-s001.zip › foods-1506731-SI.pdf]

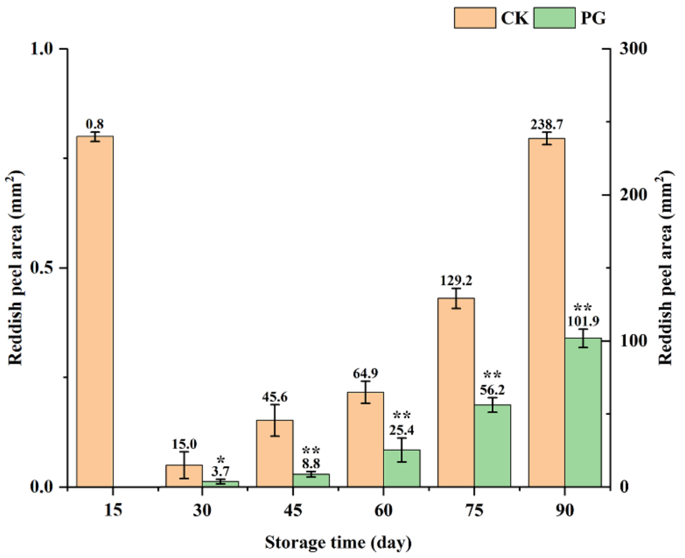

Figure S1: Effects of PG treatment on the reddish peel area of the peel. Vertical bars represent the SE of triplicate assays. The asterisks indicated significant difference between the control and PG-treated fruits during the same storage period (* P<0.05, ** P<0.01).

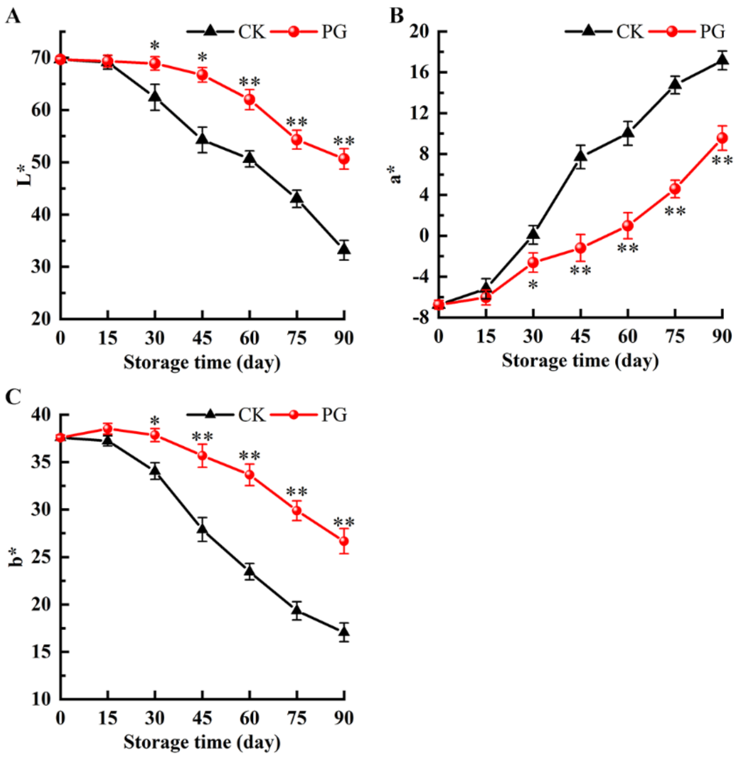

Figure S2: Effects of PG treatment on the L*(**A**), a* (**B**) and b* (**C**) of winter jujube fruits. Vertical bars represent the SE of the mean. The asterisks indicated significant difference between two treatments during the same storage period (* P<0.05, ** P<0.01).
